# ROS as a novel indicator to predict anticancer drug efficacy

**DOI:** 10.1186/s12885-019-6438-y

**Published:** 2019-12-16

**Authors:** Tarek Zaidieh, James R. Smith, Karen E. Ball, Qian An

**Affiliations:** School of Pharmacy and Biomedical Sciences, Institute of Biological and Biomedical Sciences, University of Portsmouth, Portsmouth, PO1 2DT UK

**Keywords:** Reactive oxygen species, Cisplatin, Dequalinium chloride hydrate, Drug sensitivity, Cancer biomarker

## Abstract

**Background:**

Mitochondria are considered a primary intracellular site of reactive oxygen species (ROS) generation. Generally, cancer cells with mitochondrial genetic abnormalities (copy number change and mutations) have escalated ROS levels compared to normal cells. Since high levels of ROS can trigger apoptosis, treating cancer cells with low doses of mitochondria-targeting / ROS-stimulating agents may offer cancer-specific therapy. This study aimed to investigate how baseline ROS levels might influence cancer cells’ response to ROS-stimulating therapy.

**Methods:**

Four cancer and one normal cell lines were treated with a conventional drug (cisplatin) and a mitochondria-targeting agent (dequalinium chloride hydrate) separately and jointly. Cell viability was assessed and drug combination synergisms were indicated by the combination index (CI). Mitochondrial DNA copy number (mtDNAcn), ROS and mitochondrial membrane potential (MMP) were measured, and the relative expression levels of the genes and proteins involved in ROS-mediated apoptosis pathways were also investigated.

**Results:**

Our data showed a correlation between the baseline ROS level, mtDNAcn and drug sensitivity in the tested cells. Synergistic effect of both drugs was also observed with ROS being the key contributor in cell death.

**Conclusions:**

Our findings suggest that mitochondria-targeting therapy could be more effective compared to conventional treatments. In addition, cancer cells with low levels of ROS may be more sensitive to the treatment, while cells with high levels of ROS may be more resistant. Doubtlessly, further studies employing a wider range of cell lines and in vivo experiments are needed to validate our results. However, this study provides an insight into understanding the influence of intracellular ROS on drug sensitivity, and may lead to the development of new therapeutic strategies to improve efficacy of anticancer therapy.

## Background

Mitochondria are implicated in many cellular processes such as cellular energy metabolism, cell communication, differentiation and apoptosis [1]. Mitochondrial dysfunction leads to alterations in mitochondrial structure, disruption of mitochondrial membrane potential, instability of electron transport reactions resulting in reactive oxygen species (ROS) overproduction, activation of caspase cascades and initiation of apoptosis pathway. Hence, any mitochondrial abnormality can lead to the development of several human diseases, including cancers [2]. One unique feature of mitochondria is that they contain their own genome, mitochondrial DNA (mtDNA; a small circular DNA of approximately 16,569 bp), independent of nuclear DNA [3].

Mitochondria are the primary source of intracellular ROS, a group of chemically reactive molecules containing oxygen, hydroxyl radical (•OH), superoxide anion (O_2_^−^), singlet oxygen (O_2_) and hydrogen peroxide (H_2_O_2_), as side-products of the mitochondrial electron transport chain reaction during cellular respiration [4]. ROS play important roles in cell signalling pathways such as growth, differentiation, metabolism and apoptosis [4, 5]. They are also regarded as a double-edged sword in cancer cells since low doses of ROS can promote cell proliferation and invasion, whereas excessive levels of ROS cause oxidative damage to proteins, lipids, RNA and DNA which consequently induce cell death [6, 7]. Therefore, a slight increase of ROS is associated with the initiation and progression of cancer [4, 8], but high levels of ROS can induce cell death by activating several signalling pathways resulting in cell apoptosis [6, 7]. For example, in cancer cells with *wild type* p53, DNA damage by ROS induces apoptosis in a mitochondria-dependent manner via activation of the p53/BAX signalling pathway [4]. In healthy tissues, the intracellular ROS are preserved at a steady and low level by the equilibrium between ROS production and elimination by enzymatic antioxidants such as cytoplasmic superoxide (SOD1), mitochondrial superoxide (SOD2), catalase (CAT) and glutathione (GSH) [9]. Tumour cells express lower antioxidants than normal cells, and therefore have higher ROS levels. Furthermore, defective mitochondrial oxidative metabolism in tumour cells also render higher ROS levels [9], and therefore ROS induction is a promising approach to cancer therapy [4, 8].

Despite its strong side effects, chemotherapy is still widely used in clinical practice. Many chemotherapy drugs cause cell death by a direct damage to the nucleic acids while others disrupt the redox balance within the cell. Some chemotherapeutic agents can cause an excessive accumulation of ROS either via an overproduction of ROS or by supressing their elimination in tumour cells by the antioxidant systems [10]. Cisplatin [cisplatinum or cis-diamminedichloroplatinum (II)] is one of the most commonly used chemotherapeutic agents employed in the treatment of various human cancers. It is a highly reactive molecule which forms various types of adducts by binding to DNA, RNA and proteins, and the cytotoxic effect of cisplatin is mainly due to the lesions formed within the nuclear DNA [11]. Moreover, previous studies have demonstrated that cisplatin accumulates in mitochondria and causes significant changes in mitochondrial structure and metabolic function [11, 12]. Recent reports evinced that cisplatin-induced apoptosis could be inhibited by compounds that interfere with ROS generation. These observations elucidate that the killing effect is correlated to increased ROS generation [12]. However, the clinical use of cisplatin is limited because of its severe irreversible side effects including neurotoxicity, ototoxicity and nephrotoxicity which has been reported as the main limitation of cisplatin [13]. Furthermore, the majority of current systemic cancer chemotherapeutic drugs exert their toxicity on mitochondria indirectly via different signalling pathways, and they do not localise at tumour sites efficiently and therefore can cause unwanted damage to normal tissues [2, 14].

Recently, due to their critical role in metabolism, ATP synthesis and redox status, and because of their involvements in many pathways related to the cell death, mitochondria have become one of the main interests in developing cancer treatments. Since cancer cells generally have higher levels of ROS compared to normal cells, and because of the differences in the mitochondrial membrane potential between cancer and normal cells, a direct targeting on mitochondrial functions could be an effective approach to triggering cancer-specific cell death. Delocalised lipophilic cations (DLCs), a group of small membrane permeable agents driven by negative potential across the mitochondrial membrane, accumulate in mitochondria and are more toxic to cancer cells compared to normal cells [15]. This characteristic attracts researchers to evaluate DLCs for selective cancer cell elimination [16]. Within a wide range of DLCs, dequalinium (DQA) has been reported to demonstrate a potent anticancer activity in vitro and in vivo in different malignancies [14]. Several studies have suggested that the cytotoxicity mechanism of DQA is related to mitochondrial dysfunction due to the damage of mitochondrial DNA and the inhibition of mitochondrial complex I [17]. It has also been reported that DQA causes cell death in the HeLa cells by selective depletion of mtDNA [18]. Moreover, it has been postulated that DQA induces human leukaemia cell death by affecting the redox balance [19], and another study showed that DQA caused oxidative stress and apoptosis in a human prostate cancer cell line [20].

Due to the merit of mitochondria-targeting therapy, the combination of conventional chemotherapy drugs such as cisplatin with mitochondria-targeting agents may offer a promising strategy for enhanced anticancer therapy [21]. Furthermore, mitochondrial DNA copy number (mtDNAcn) per cell is preserved within a stable range to achieve the required energy of the cell and hence ensure normal physiological functions. It ranges from 10^3^ to 10^4^ according to the population and cell type. Such variations also reflect the imbalance between ROS production and the antioxidant capacity, so mtDNAcn has been considered as a potential diagnostic and prognostic biomarkers for several cancer types [22].

This study aimed to investigate the link between mtDNAcn and baseline intracellular ROS level in untreated cancer cells, as well as how baseline ROS level might influence cells’ response to ROS-stimulating therapy. The potential synergistic effect of cisplatin and dequalinium chloride in killing cancer cells was also assessed.

## Methods

### Cell culture

The four cancerous (Ishikawa/endometrium, MDA-MB-231/breast, Caco-2/colon, PC-3/prostate) and one normal (PNT-2/prostate) cell lines were obtained from the departmental cell bank at the University of Portsmouth. All cell lines were originally purchased from the European Collection of Authenticated Cell Cultures/ECACC (Ishikawa, MDA-MB-231, Caco-2, PNT-2) or the American Type Culture Collection/ATCC (PC-3). Ishikawa (Cat. 99040201), Caco-2 (Cat. 86010202) and PNT-2 (Cat. 95012613) were purchased in 2015; MDA-MB-231 (Cat. 92020424) and PC-3 (Cat. CRL-1435) were purchased in 2013. Cells were maintained in required media (Additional file 1: Table S1) and harvested at 90% confluence for the downstream assays. All cell lines were authenticated to confirm their identities by STR (short tandem repeat) DNA profiling using the PowerPlex 16 HS System (Promega, Southampton, UK) and screened for mycoplasma contamination using the PCR Mycoplasma Test Kit (PromoCell, Heidelberg, Germany). Cell authentication and mycoplasma detection tests were conducted in the beginning, the middle and towards the end of the investigation according to the established protocols in our laboratories.

### Drug treatment

Stock solutions of cisplatin (CDDP) and dequalinium chloride hydrate (DQA) (Sigma, Dorset, UK) were prepared at 100 mM in DMSO and 2 mM in distilled water, respectively. Both drugs were added to the cells in various concentrations and incubated for 24 h to determine their IC50s (IC50: the half maximal inhibitory concentration) which were used in all subsequent experiments. N-Acetyl-L-cysteine (NAC) (Sigma), a powerful antioxidant, was dissolved in distilled water at the concentration of 100 mM shortly before each experiment, and the pH was adjusted to 7.4 before diluting the solution to the working concentration (10 mM) with complete cell culture medium. Cells were pre-incubated with NAC (10 mM) for 1 h prior to the CDDP and DQA treatments. All experiments related to NAC followed the same procedure as described in the sections below.

### Cell viability assay

Cell viability was measured colorimetrically using 3-(4,5-dimethylthiazol-2-yl)-5-(3-carboxymethoxyphenyl)-2-(4-sulfophenyl)-2H-tetrazolium (MTS) (CellTiter 96 Aqueous) (Promega). Briefly, 90 μl of cell suspension containing 10,000 cells was added in each well of the 96-well plate and incubated for 24 h. Cells were then treated with the 10 × drug solution (10 μl) for the desired amount of time. At the end of the experiment, the medium was replaced with 100 μl of fresh medium containing MTS (final concentration - 0.3 mg/ml) and incubated for 90–180 min according to the optimised protocol for each cell line. Absorbance was measured using the microplate reader (Multiskan® GO) at 490 nm. IC50 was calculated as the concentration of the drug that caused a 50% loss of metabolic activity.

### Combination index (CI) for synergism assessment

The synergistic effect of CDDP and DQA was assessed by the CI values calculated at different drug combinations according to the median-effect principle of the Chou and Talalay method, using the CompuSyn Software 1.4 [23]. The CI values indicate how drug combinations influence the therapeutic efficacy, i.e. CI > 1 – Antagonistic; CI = 1 – Additive; CI < 1 – Synergistic.

### ROS assays

Baseline intracellular ROS levels were measured in a 96-well plate format using the Cellular Reactive Oxygen Species Detection Assay Kit (Abcam, Cambridge, UK) based on a fluorogenic dye, H2DCFDA, according the manufacturer’s protocol. Cells (25,000/well) were seeded in the 96-well black-wall plate (Corning, NY, USA) overnight prior to the experiments. In the following day, cells were washed with HBSS (150 μl; Gibco, ThermoFisher Scientific, Loughborough, UK), then staining buffer (100 μl, 20 μM of H2DCFDA in HBSS) was added to each well, and the plate was incubated for 40 min at 37 °C. The cells were then washed with HBSS, and HBSS (100 μl) was added to each well. Fluorescence was measured using the microplate reader (POLARstar Omega) at 485 nm (excitation) and 535 nm (emission). For determining intracellular ROS levels upon treatments, the staining buffer was added and then the plate was incubated with the treatment solution for the desired amount of time before fluorescence was measured.

To measure mitochondrial superoxide, the same procedure was conducted as described above using a mitochondrial ROS specific dye, MitoSOX™ (Life Technologies, ThermoFisher Scientific), instead of H2DCFDA. The cells were incubated with 5 μM of MitoSOX in HBSS. Fluorescence was measured using the same microplate reader at 510 nm (excitation) and 580 nm (emission).

### Mitochondrial membrane potential assay

MMP was measured by staining the cells with the JC-10 fluorescent dye (Enzo Life Sciences, Exeter, UK) according the manufacturer’s protocol. Briefly, 25,000 cells were seeded in each well of the 96-well black-wall plate overnight prior to the experiments. The next day, cells were treated with required concentrations of the drugs for the desired amount of time. Following drug treatments, cells were washed with HBSS, then staining buffer (100 μl, 20 μM of JC-10 in HBSS) was added to each well and the plate was incubated for 40 min at 37 °C. Cells were then washed again with HBSS and HBSS (100 μl) was added to each well before reading the plate. Red and green fluorescence were measured using the microplate reader (POLARstar Omega) at 540 nm (excitation) / 590 nm (emission) and 490 nm (excitation) / 525 nm (emission), respectively.

### DNA extraction and measurement of mtDNAcn by SYBR Green real-time PCR

Total DNA was isolated from untreated and treated (24-h incubation) cells using the QIAmp DNA Mini Kit (QIAGEN, Hilden, Germany) according to the manufacture’s protocol. Relative quantification of mtDNAcn was measured using the QuantiTect SYBR Green PCR kit (QIAGEN) and run on a LightCycler® 96 System (Roche, Basel, Switzerland) according to previously published method [24]. The relative quantity of mtDNA content in each sample was calculated by normalising the copy number of mtDNA against that of the housekeeping gene, *β*-actin. Three independent experiments were carried out and all samples were run in triplicates in each experiment.

### RNA extraction, reverse transcription and TaqMan real-time PCR

Total RNA was isolated from untreated and treated (24-h incubation) cells using the RNeasy Mini Kit (QIAGEN) according to the manufacturer’s protocol. In the last step, RNA was eluted with RNA–free sterile water (40 μl). cDNA was synthesised using the High-Capacity cDNA Reverse Transcription Kit (Applied Biosystems, California, USA) with 1 μg of RNA template in a 20-μl reaction.

TaqMan real-time PCR was performed using the pre-designed assays (Integrated DNA Technologies, Belgium) to measure expression levels of antioxidant genes including *SOD1*, *SOD2* and *CAT* (Additional file 1: Table S2). The experiments were performed using the FastStart Essential DNA Probes Master (Roche) and run on a LightCycler® 96 System (Roche). The amplification procedure entailed 45 cycles of 95 °C for 10 s followed by 60 °C for 30 s. For each reaction, GAPDH was utilised as the endogenous control gene. The average mRNA fold change in drug-treated samples was normalised against untreated samples using the 2^-∆∆CT^ method. Three independent experiments were carried out and all samples were run in triplicates in each experiment.

### Western blotting

WB was performed to measure the BCL-XL and released cytochrome *c* proteins from mitochondria according to the established protocols in our laboratories. Briefly, protein extracts (20 μg) were resolved by SDS-PAGE electrophoresis, and the blots were visualised using a high-sensitivity CCD camera imaging platform (Chemidoc MP; Bio-Rad, Watford, UK). ImageJ software was used for the densitometric quantification of the western blot bands. The primary and secondary antibodies employed in the experiments are listed in Additional file 1: Table S3.

### Caspase activity assay

Caspase-3/7 activity was measured using the Caspase-Glo 3/7 reagent from the ApoTox-Glo™ Triplex Assay kit (Promega). According to the manufacture protocol, 90 μl of cell suspension containing 20,000 cells was added in each well of the 96-well black-wall plate and incubated overnight. Cells were then treated with drugs for 24 h. At the end of the experiment, fresh Caspase-Glo 3/7 reagent (100 μl) was added to each well, and the plate was incubated for 60 min at room temperature. Luminescence was then measured using the microplate reader (POLARstar Omega).

### Statistical analyses

Data were analysed with the GraphPad Prism 8.0 program (Graphpad software, CA, USA) and presented as the mean ± SEM of at least three separate experiments. Specific statistical tests used to analyse various data sets are described in the associated figure legends. Differences between groups were considered statistically significant according the following criteria: *p < 0.05, **p < 0.01, ***p < 0.001 and ****p < 0.0001.

## Results

### Correlation between baseline ROS level, mtDNAcn and drug sensitivity in cancer cells

**Fig. 1:**
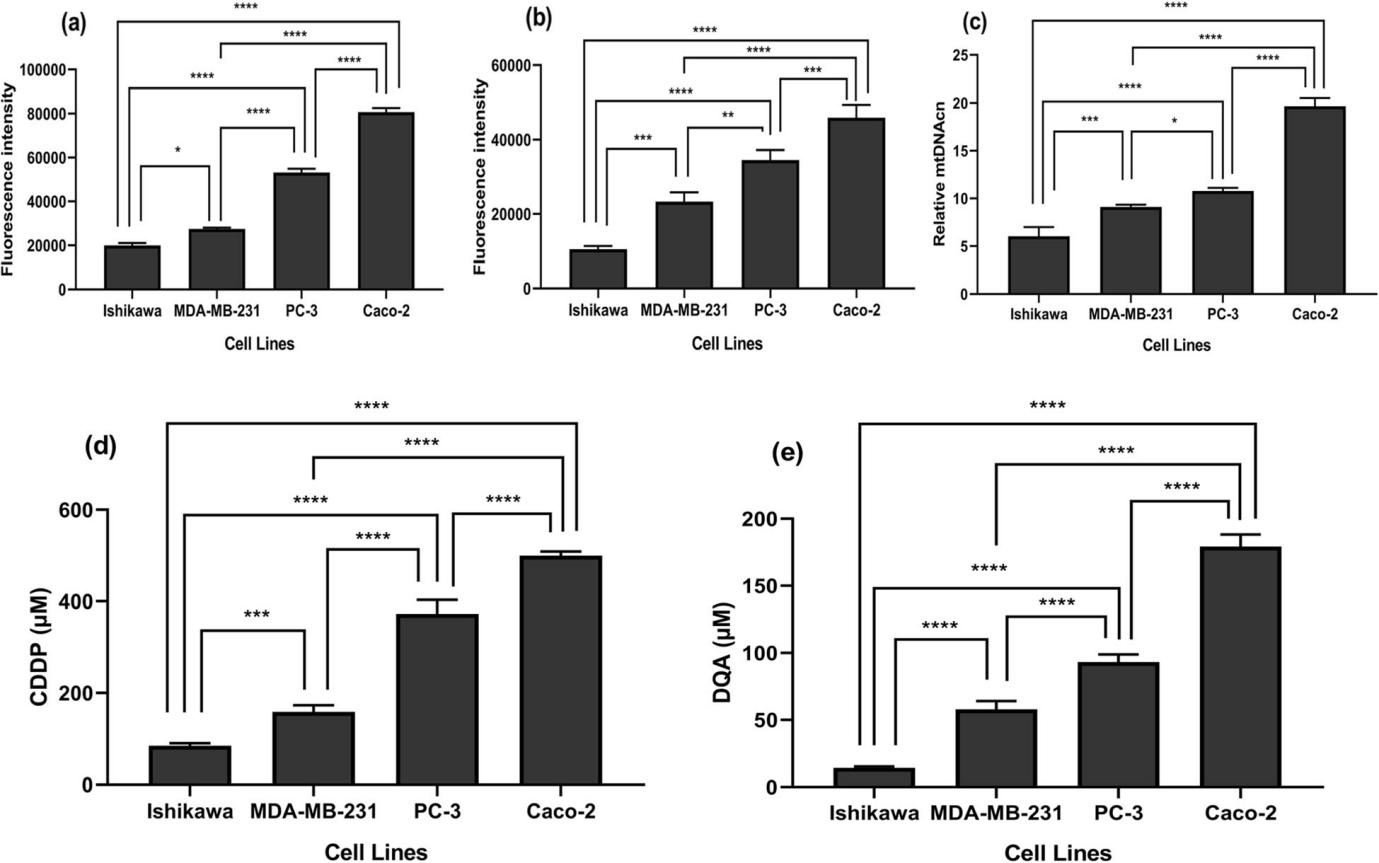
Correlation between the baseline ROS level, mtDNA copy number and drug sensitivity. Intracellular ROS (**a**) and mitochondrial superoxide (**b**) levels of the Ishikawa, MDA-MB-231, PC-3 and Caco-2 cell lines are represented by the fluorescence intensity of H2DCFDA and MitoSOX respectively. (**c**) Relative content of mtDNA in Ishikawa, MDA-MB-231, PC-3 and Caco-2 normalised against the house keeping gene (*β*-actin). The columns represent the relative mtDNA copy numbers of the cell lines. Comparison of the CDDP IC50s (**d**) and the DQA IC50s (**e**) amongst the 4 cell lines. The columns represent the CDDP IC50s and the DQA IC50s in the cell lines. Data are mean ± SEM (*N* = 3 separate experiments) in all of the figure panels; *p* values were calculated using one-way ANOVA with Tukey multiple comparison post-hoc analysis; *p < 0.05, **p < 0.01, ***p < 0.001 and ****p < 0.0001

The H2DCFDA and MitoSOX assays results indicated that the Caco-2 cell line had the highest baseline ROS and mitochondrial superoxide levels whereas Ishikawa had the lowest (*p <* 0.0001; Fig. 1a & b). The baseline ROS level correlated positively to the mtDNA copy number amongst the cell lines, as shown in Fig. 1c that the Caco-2 cell line had the highest mtDNA copy number whereas Ishikawa the lowest (*p <* 0.0001). To evaluate the drug sensitivity of all the cell lines towards CDDP and DQA, cells were treated with various concentrations of the drugs separately for 24 h. The results showed that the Ishikawa cells were significantly more sensitive to both drugs compared to other cell lines whereas the Caco-2 cells were significantly more resistant (Fig. 1d & e). The IC50s of the cell lines were calculated, and the results showed that the IC50s of CDDP were 84.96 ± 3, 158.9 ± 8.2, 372.7 ± 17.5 and 499.5 ± 15.1 μM for Ishikawa, MDA-MB-231, PC-3 and Caco-2, respectively, whereas the IC50s of DQA were 14.24 ± 0.59, 57.85 ± 3.59, 93.31 ± 3.21 and 179.2 ± 5.2 μM for the above cell lines. Based on these observations, the Caco-2 and Ishikawa cell lines were chosen to represent the most resistant and sensitive cell lines respectively in the downstream experiments to investigate the underlying killing mechanisms that might be influenced by baseline intracellular ROS levels.

### Synergistic effect of CDDP and DQA observed in Ishikawa and Caco-2

**Fig. 2:**
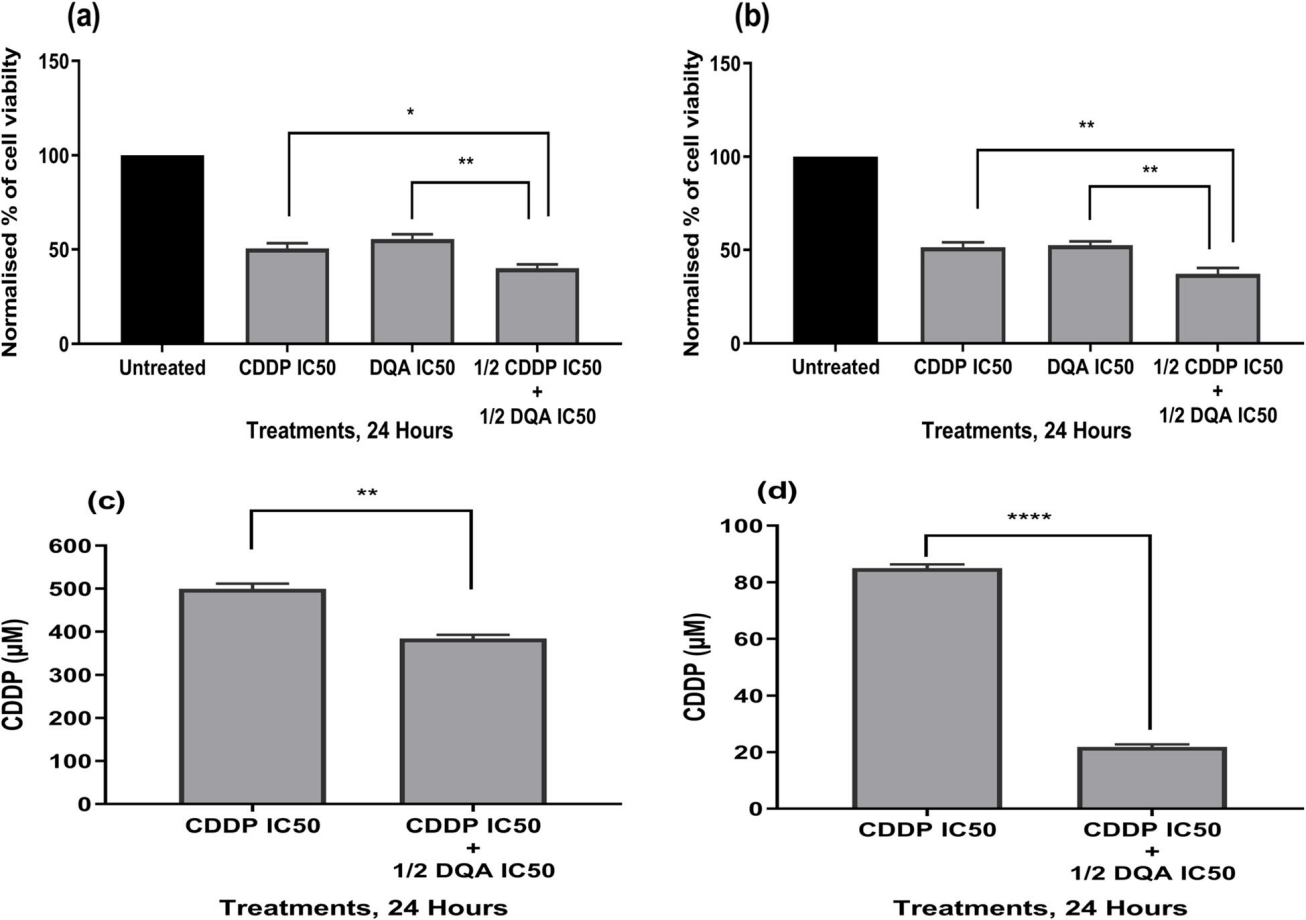
Comparison of Caco-2 (**a**) and Ishikawa (**b**) cell viability upon treatments with CDDP and DQA at their IC50 concentrations, and a combination of ½ IC50 of both drugs at 24 h. The columns represent cell viability under various treatment conditions normalised against the untreated controls. Data are mean ± SEM (*N* = 3 separate experiments); *p* values comparing single and combined treatments were calculated using one-way ANOVA with Tukey multiple comparison post-hoc analysis; *p < 0.05, **p < 0.01. **c** Comparison between the CDDP IC50 (499.5 μM) in single drug treatment and the new CDDP IC50 (384 μM) in combination with ½ DQA IC50 in the Caco-2 cells. **d** Comparison between the CDDP IC50 (84.9 μM) in single drug treatment and the new CDDP IC50 (21.8 μM) in combination with ½ DQA IC50 in the Ishikawa cells. Data are mean ± SEM (*N* = 3 separate experiments); *p* values comparing single and combined treatments were calculated using a two-tailed t-test; **p < 0.01 and ****p < 0.0001

Incubation with a combined treatment of CDDP and DQA at half of their IC50 concentrations resulted in a marked reduction of cell viability in both Caco-2 and Ishikawa cells, compared to the cells treated with a single drug (either CDDP or DQA) at its IC50 (Fig. 2a & b). Moreover, treating cells with various concentrations of CDDP in combination with DQA at half of its IC50 (i.e. 89.6 μM for Caco-2 and 7.2 μM for Ishikawa) resulted in a significant decrease of CDDP IC50 for the Caco-2 (384 ± 8.3 μM) (*p <* 0.01) and Ishikawa (21.8 ± .91 μM) (*p <* 0.0001) cells (Fig. 2c & d). Data from the CompuSyn analysis also indicated synergistic effects of the compounds within the concentration range of 5–250 μM and 5–1000 μM for CDDP in Caco-2 and Ishikawa, respectively (Additional file 1: Table S4).

### Intracellular ROS and mitochondrial superoxide levels increased upon treatments

**Fig. 3:**
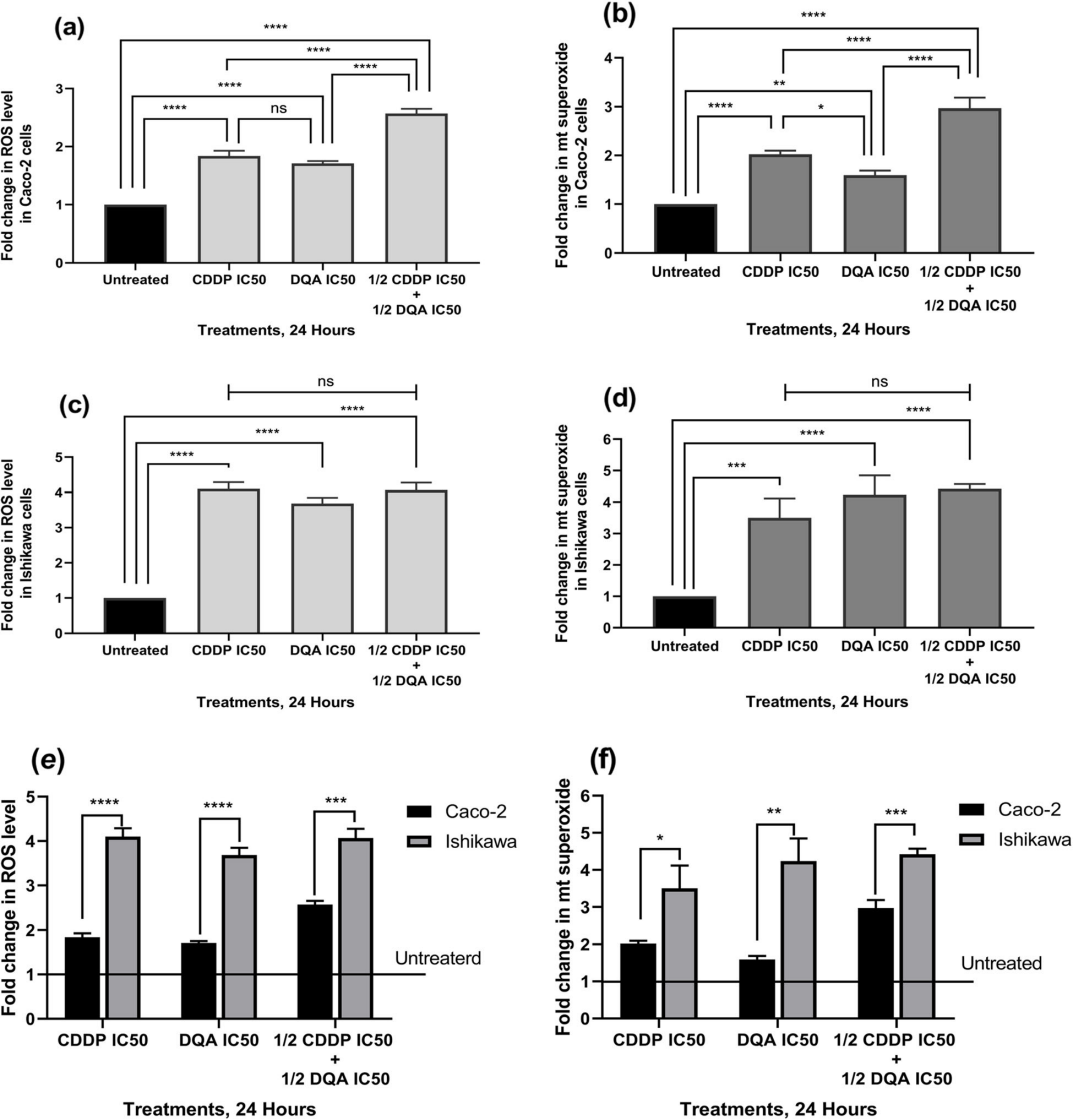
Increases of intracellular ROS level and mitochondrial superoxide in the Caco-2 (**a** & **b**) and Ishikawa (**c** & **d**) cells upon treatments at 24 h. The columns represent the fold changes of ROS and mitochondrial superoxide normalised against the untreated controls. Data are mean ± SEM (*N* = 3 separate experiments); *p* values comparing treated and untreated cells were calculated using one-way ANOVA with Tukey multiple comparison post-hoc analysis; ns, not significant, *p < 0.05, **p < 0.01, ***p < 0.001 and ****p < 0.0001. **e** Comparison of intracellular ROS level changes upon treatments between the Caco-2 and Ishikawa cells at 24 h. **f** Comparison of mitochondrial superoxide changes upon treatments between the Caco-2 and Ishikawa cells at 24 h. Data are mean ± SEM (*N* = 3 separate experiments); *p* values comparing the Caco-2 and Ishikawa cells were calculated using a two-tailed t-test; *p < 0.05, **p < 0.01, ***p < 0.001 and ****p < 0.0001

Continuous exposure to CDDP and DQA at their IC50 concentrations and to the combination of both drugs at half of their IC50 concentrations for 24 h resulted in significant increases of general intracellular and mitochondrial ROS levels in both Caco-2 and Ishikawa cells in a time-dependent manner. Significant increases in intracellular ROS were observed in the Caco-2 cells at 5 h following initial exposures to CDDP and the combined therapy (*p <* 0.01) (Additional file 1: Figure S1a & c), and at 7 h following DQA treatment (*p <* 0.001) (Additional file 1: Figure S1b). ROS levels increased continuously up to 24 h in the Caco-2 cells under all treatments. Earlier increases of intracellular ROS were observed in the Ishikawa cells, i.e. at 3 h following initial exposures to CDDP and DQA (*p <* 0.001) (Additional file 1: Figure S1d & e), and at 1 h with the combined therapy (*p <* 0.05) (Additional file 1: Figure S1f). Parallel increases in mitochondrial superoxide were observed in both cell lines (Additional file 1: Figure S1). Furthermore, greater increases in intracellular ROS and mitochondrial superoxide were observed with the combined therapy compared to single treatment of CDDP or DQA in the Caco-2 cells at 24 h (*p* < 0.0001; Fig. 3a & b). On the contrary, ROS production was equally elevated in the Ishikawa cells under all treatment conditions (Fig. 3c & d). However, the Ishikawa cells showed markedly higher increases in intracellular ROS and mitochondrial superoxide upon treatments compared to Caco-2 (Fig. 3e & f).

### Cell viability and mitochondrial membrane potential reduction along the same timeline as increased ROS generation

A decrease in cell viability was observed following the treatments in both cell lines in a time-dependent manner (Additional file 1: Figure S2), and this change correlated with a decrease in MMP (Additional file 1: Figure S3). Our data showed that CDDP noticeably depolarised mitochondrial potential at 1 and 3 h in Ishikawa and Caco-2, respectively, and MMP continued to reduce up to 24 h in both cell lines. Interestingly, cells treated with DQA and the combined therapy showed significantly greater mitochondrial depolarisation at the 1-h time point compared to the CDDP treatment in both cell lines, predominantly in Ishikawa. As described above, ROS generation (Additional file 1: Figure S1) was also time-dependent within the 24-h drug treatment period.

### MtDNAcn decreased upon treatments

Incubation with CDDP, DQA and their combination for 24 h resulted in a marked reduction of mtDNAcn in both cell lines (Additional file 1: Figure S4), with the copy number at its lowest value in Caco-2 undergone the combined treatment (*p <* 0.0001; Additional file 1: Figure S4a).

### Cancer-preferential uptake of DQA

The JC-10 assay results indicated that the PC-3 cells (prostate cancer) had much higher MMP compared to their non-cancerous counterparts, the PNT-2 cells (Additional file 1: Figure S5a). Exposing to 10 μM of DQA resulted in a more rapid mitochondrial depolarisation in PC-3 compared to PNT-2 during a 3-h incubation (Additional file 1: Figure S5b).

### ROS as the main contributor in cell death

**Fig. 4:**
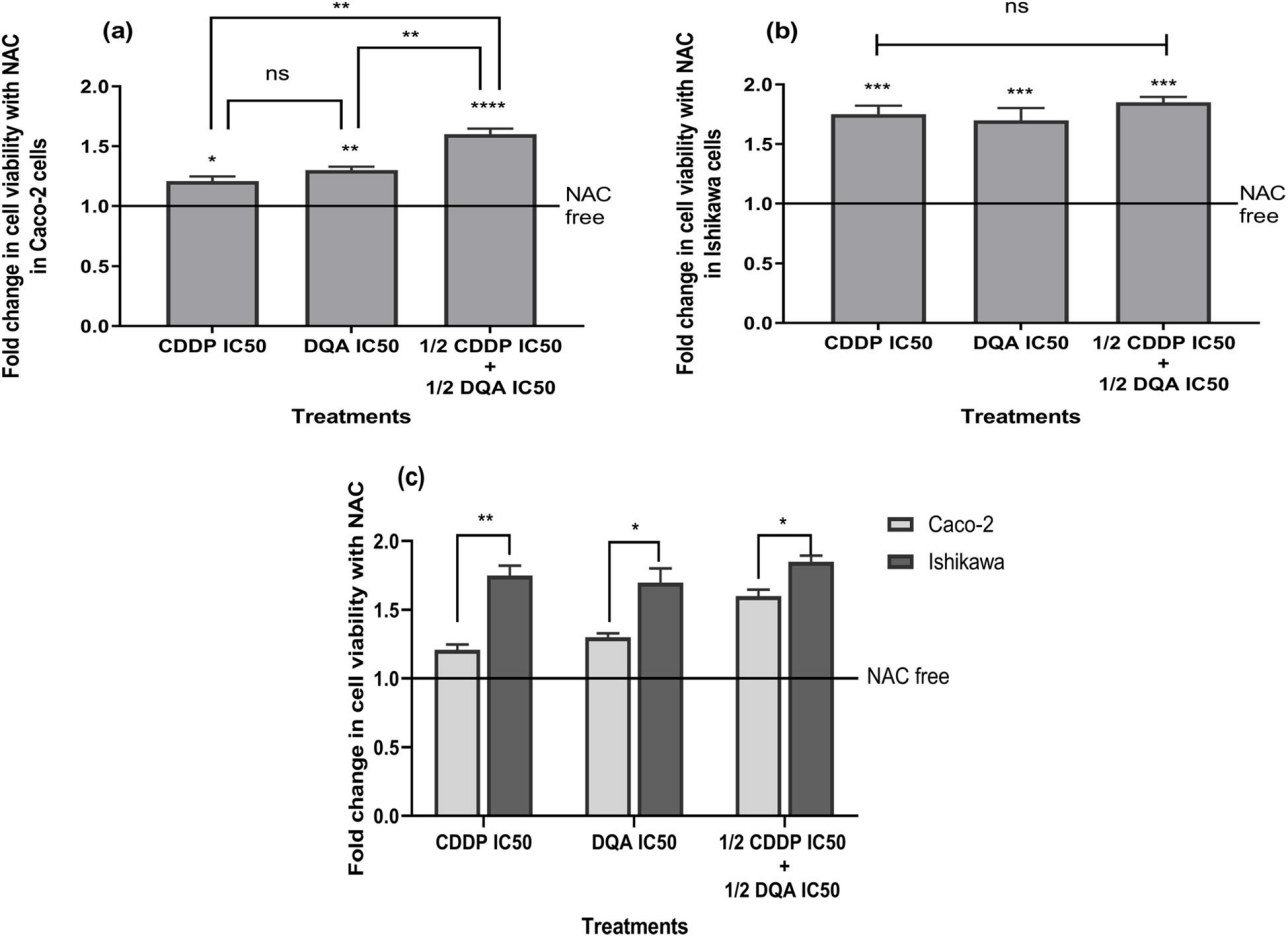
Increased cell viability of Caco-2 (**a**) and Ishikawa (**b**) upon pre-incubation with NAC (10 mM) prior to drug treatments. The columns represent the fold changes in cell viability normalised against NAC-free treatment. Data are mean ± SEM (*N* = 3 separate experiments); *p* values comparing NAC-free and NAC-pre-incubated cells were calculated using one-way ANOVA with Tukey multiple comparison post-hoc analysis; ns, not significant, *p < 0.05, **p < 0.01, ***p < 0.001 and ****p < 0.0001. **c** Comparison of the changes in cell viability between the Caco-2 and Ishikawa cells upon pre-incubation with NAC. Data are mean ± SEM (*N* = 3 separate experiments); *p* values comparing Caco-2 and Ishikawa were calculated using two-tailed t-test; *p < 0.05 and **p < 0.01

Cell death induced by CDDP, DQA and the combined treatment was significantly reduced by pre-incubation with NAC (10 mM) (Fig. 4a & b). Those data showed that cell death attenuation with the combined therapy in Caco-2 was more significant than that with the single treatments, whereas the attenuation in Ishikawa was comparable amongst different treatments. Furthermore, cell death attenuation upon pre-incubation with NAC was more significant in Ishikawa compared to Caco-2 (Fig. 4c).

### Gene expression levels of anti-oxidants altered upon treatments

**Fig. 5:**
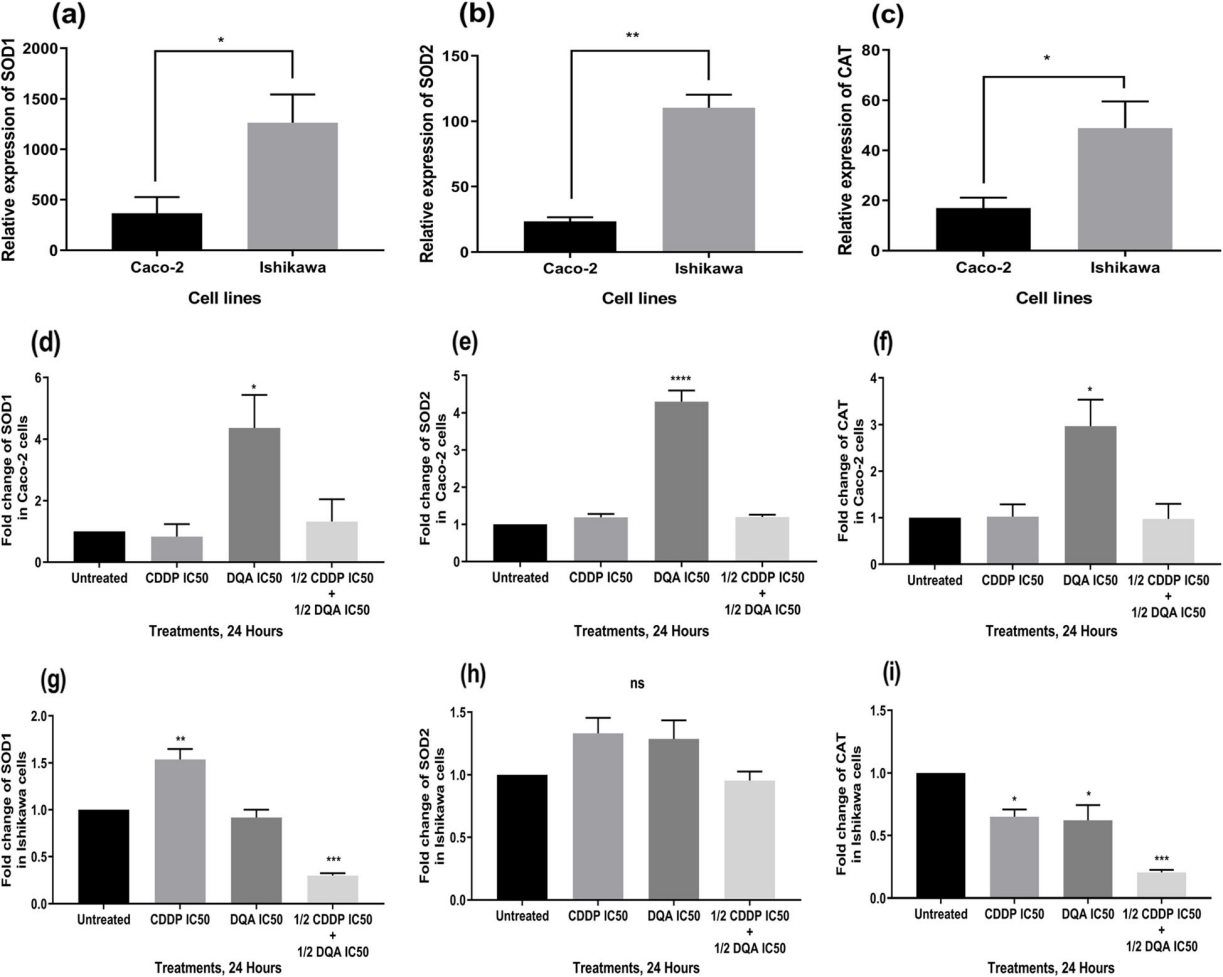
Comparison of the relative expression levels of the *SOD1* (**a**), *SOD2* (**b**) and *CAT* (**c**) genes between the untreated Caco-2 and Ishikawa cells. The columns represent the relative expression levels of the genes using TaqMan qPCR. Data are mean ± SEM (*N* = 3 separate experiments); *p* values comparing the Caco-2 and Ishikawa cells were calculated using two-tailed t-test; *p < 0.05 and **p < 0.01. Gene expression level changes in the Caco-2 (**d-f**) and Ishikawa (**g-i***)* cells upon treatments at 24 h. The columns represent the fold changes of the relative expression levels of the genes normalised against the untreated controls. Data are mean ± SEM (*N* = 3 separate experiments); *p* values comparing treated and untreated cells were calculated using one-way ANOVA with Tukey multiple comparison post-hoc analysis; ns, not significant, *p < 0.05, **p < 0.01, ***p < 0.001 and ****p < 0.0001

The relative baseline expression levels of the three antioxidant genes, *SOD1*, *SOD2* and *CAT*, were significantly lower in the Caco-2 cells compared to the Ishikawa cells (Fig. 5a-c). Interestingly, all genes were significantly upregulated in Caco-2 in the presence of DQA but no significant changes were observed with CDDP or the combined therapy for these three genes (Fig. 5d-f). In Ishikawa, *SOD1* was significantly upregulated in the presence of CDDP, whereas a significant downregulation in the combined therapy was observed (Fig. 5g). *SOD2* did not show any significant changes upon treatments (Fig. 5h) while *CAT* was significantly downregulated in the presence of all treatments with a further downregulation in the combined therapy (Fig. 5i).

### Apoptosis events revoked upon elimination of ROS

**Fig. 6:**
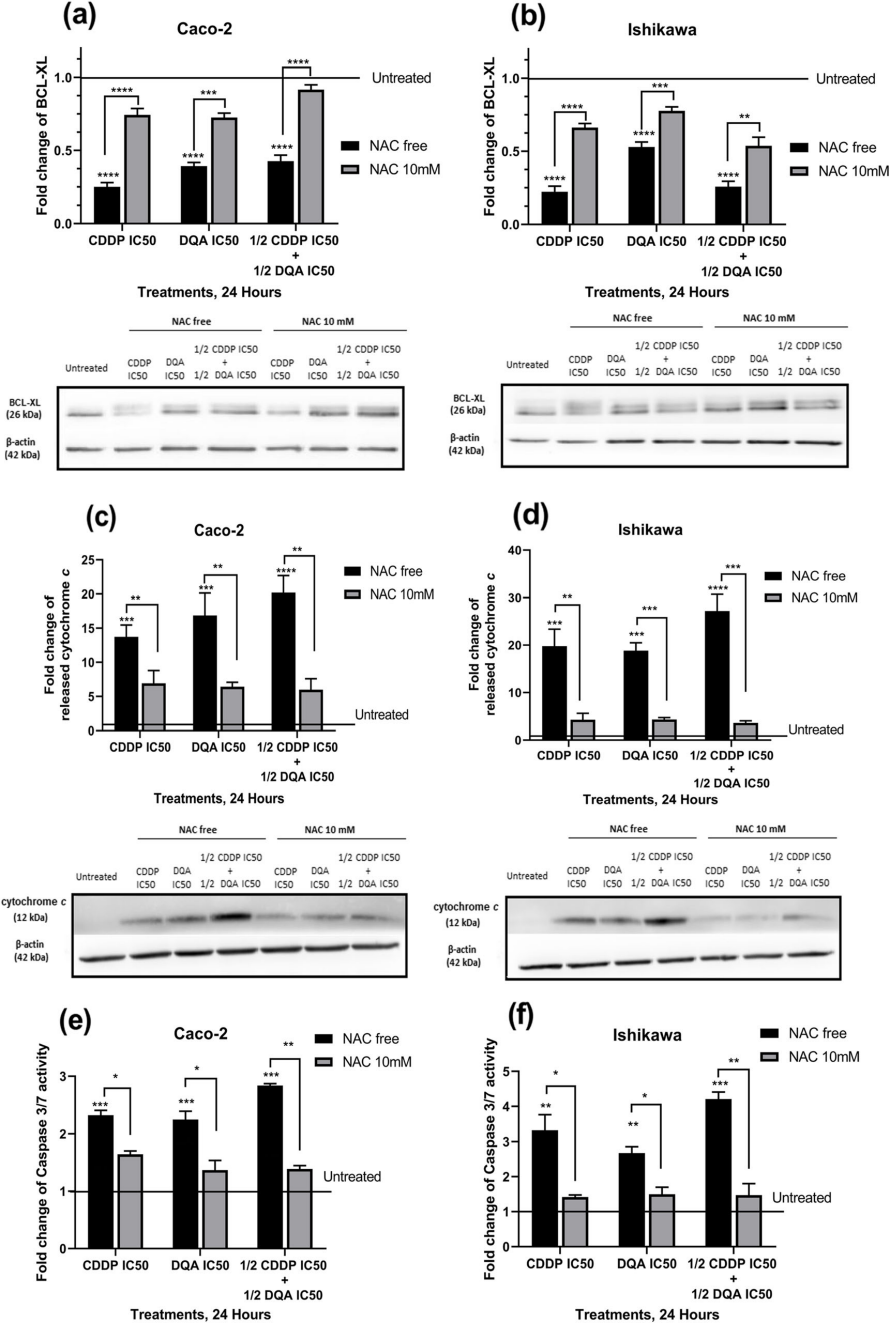
Western blots showing changes in the protein levels of BCL-XL (**a** & **b**) and release of cytochrome *c* (**c** & **d**) in the Caco-2 and Ishikawa cells upon treatments at 24 h and the effect of NAC pre-incubation in attenuating theses changes in both cell lines. The columns represent the fold changes of the protein levels normalised against the loading control protein, β-Actin. Data are mean ± SEM (*N* = 3 separate experiments); *p* values comparing CDDP/DQA-treated and -untreated cells without NAC pre-incubation were calculated using one-way ANOVA with Tukey multiple comparison post-hoc analysis; ***p < 0.001 and ****p < 0.0001. *p* values comparing NAC-free and NAC-pre-incubated cells undergone the same CDDP/DQA treatments were calculated using a two-tailed t-test; **p < 0.01, ***p < 0.001 and ****p < 0.0001. Caspase activity assay showing changes in the caspase-3/7 activity in the Caco-2 (**e**) and Ishikawa (**f**) cells upon treatments for 24 h and the effect of NAC pre-incubation in attenuating theses changes in both cell lines. The columns represent the fold changes of the caspase-3/7 activity normalised against the untreated cells. Data are mean ± SEM (*N* = 2 separate experiments); *p* values comparing CDDP/DQA-treated and -untreated cells without NAC pre-incubation were calculated using one-way ANOVA with Tukey multiple comparison post-hoc analysis; **p < 0.01 and ***p < 0.001. *p* values comparing NAC-free and NAC-pre-incubated cells undergone the same CDDP/DQA treatments were calculated using a two-tailed t-test; *p < 0.05 and **p < 0.01

To determine whether the observed cell death was due to ROS-induced apoptosis upon the treatments, apoptosis-associated proteins were measured in cells with or without the pre-incubation of NAC. For the cells without NAC pre-incubation, our western blotting data showed significant decreases in the anti-apoptotic protein, BCL-XL, upon treatments in both cell lines (*p <* 0.0001; Fig. 6a & b), whereas the release of cytochrome *c* was significantly increased upon the treatments (*p* < 0.0001; Fig. 6c & d). Caspase activation assay also showed a significant activation of caspase-3/7 upon the treatments (Fig. 6e & f). All above events were significantly attenuated by NAC pre-incubation.

## Discussion

Several previous studies have demonstrated that various types of cancer cell lines have elevated levels of ROS compared to normal cell lines [25–27]. Our own ROS assays conducted with one paired normal-cancer cell lines to verify this previous observation confirmed it to be the case (data not shown). Cancer cells also have various levels of mtDNAcn which are strictly correlated to the demand of ATP generated by OXPHOS, “the major cause of ROS production” [26, 27]. Indeed, our data showed a positive correlation between the baseline ROS level and the mtDNAcn amongst 4 human cancer cell lines of different tissue origins, which confirmed the previous observation that cells containing larger mtDNA copy numbers also have higher levels of ROS.

It has been postulated that cisplatin increases the generation of intracellular ROS which may cause damage to DNA, proteins and lipids leading to apoptosis [28]. Several previous studies also related the mechanism of the antitumour effect of dequalinium to an impairment of mitochondrial function and the associated ROS generation [2, 14, 19]. Therefore, the potential link between the baseline ROS levels in cancer cells and their drug sensitivity towards cisplatin and dequalinium was investigated in the present study. Our results indicated that cancer cells with high baseline ROS levels were significantly more resistant to both cisplatin and dequalinium compared to cells with low ROS levels. Therefore, baseline ROS levels could be utilised to predict drug response of cancer cells. As discussed above, the good correlation between mtDNAcn and baseline intracellular ROS level in naïve cancer cells also means that mtDNAcn could be a potential biomarker to indicate drug sensitivity. Furthermore, our western blotting and caspase3/7 activity data proved that the mode of action of the two compounds was indeed through ROS-induced apoptosis. It is worth mentioning that comparable mitochondrial content levels were detected in Caco-2 and Ishikawa using MitoTracker™ Red CMXRos in our study (data not shown). This finding suggests that baseline ROS level (either intracellular or MitoSOX) might be more indicative of drug response compared to the population of the organelles in the cells. Since the Caco-2 cells had the highest level of mtDNAcn amongst the 4 cancer cell lines, we speculate that the average copy number of mtDNA per mitochondrion must be far greater in a single Caco-2 cell than that in a single Ishikawa cell (it is widely acknowledged that each mitochondrion can contain 2–10 copies of mtDNA). In addition, our data suggest that MitoSOX production in the cell may be influenced by the cell’s mtDNAcn rather than its mitochondrial population.

Currently, cisplatin has limited clinical use due to its severe side effects [29]. Therefore, it is plausible to apply a lower dose of cisplatin in combination with another anticancer agent in order to reduce the side effects of cisplatin while enhancing the therapeutic efficacy. In this study, significant synergistic effects of CDDP and DQA were observed in both Caco-2 and Ishikawa cells. Those results were in alignment with previous findings that mitochondria are promising target for anticancer therapy [30]. Furthermore, our data clearly suggest that combining mitochondria-targeting agents could provide improved efficacy while reducing side effects of conventional therapy in treating cancer.

Interestingly, the present study revealed a significant difference between the Caco-2 and Ishikawa cells in terms of their response and behaviour to the treatments at cellular and molecular levels. ROS production upon drug exposure was significantly elevated in Ishikawa compared to Caco-2, and the onset of ROS production was far more rapid in Ishikawa than that in Caco-2. In addition, the Caco-2 cells responded most significantly to the combined therapy, whereas similar levels of increased ROS generation were observed in the Ishikawa cells across all treatments. These results suggested that CDDP, DQA and their combination could stimulate more ROS production in cells with relatively low baseline levels of intracellular ROS. As elevated ROS can trigger cell death, this explains the significantly higher sensitivity of the Ishikawa cells towards those treatments compared to the Caco-2 cells. For cells with relatively high baseline ROS levels, the combined treatment of CDDP and DQA could have a greater impact on ROS production compared to the single treatment of the compounds. This is in line with our aforementioned observation that combining CDDP with DQA had a synergistic effect in killing cancer cells. Since cancer cells with higher baseline ROS levels could be more resistant to CDDP, combining CDDP and DQA might warrant a new strategy to tackle such resistance issues in the future.

A previous study showed that dequalinium induced a selective depletion of mitochondrial DNA in carcinoma cells [18]. Other reports also demonstrated that cisplatin accumulated in mitochondria formed adducts in mtDNA that interfered with mtDNA transcription and replication, and caused ATP deficiency which then led to cell death [11, 31, 32]. Our results showed significant decreases of mtDNAcn in both cancer cell lines upon the treatments and the relative mtDNAcn value reached the lowest in samples that had undergone the combined therapy. Such mtDNA depletion upon treatments would reduce the expression levels of mtDNA-encoded complex subunits of the electron transport chain (ETC) and cause more leakage of electrons during OXPHOS, consequently leading to an excessive generation of ROS.

Since mitochondrial membrane depolarisation is an early key feature of cell death and also could affect respiration and increase ROS generation [33], changes in the mitochondrial membrane potential upon treatments were examined in the present study. Indeed, depolarisations occurred in both cell lines upon treatments in a time-dependent manner, which indicated that both CDDP and DQA affected the mitochondrial membrane potential and interfered with ROS generation. A far more rapid and overwhelming depolarisation upon DQA treatment and its combination with CDDP was observed in both cell lines compared to the MMP changes observed in cells treated with CDDP alone (Additional file 1: Figure S3). It should be noted that unlike the synergistic effects on ROS production (Additional file 1: Figure S1) and cytotoxicity (Additional file 1: Figure S2), the combined treatment did not have a synergistic effect on mitochondrial depolarisation in both cell lines. In fact, although the combined treatment had a profound impact on mitochondrial membrane potential, cells treated with DQA alone showed the most rapid and significant depolarisation upon the treatment. This could be attributed to the positive charge of DQA which led to its prompt and excessive accumulation within the mitochondria, consequently neutralising the negative electric potential on the inner mitochondrial membrane at a speedy pace. Such swift changes in MMP were observed in our time course experiments. The impact on MMP was less severe from the combined treatment compared to that from DQA alone during the early phase of the time course particularly at the 1-h and 3-h time points. This could be due to the fact that only half of the DQA dosage was administrated to the cells in the combined treatment, which affected the speed and extent of depolarisation in those cells. Data from our recent time course experiments using another DLC, triphenylphosphine, to replace DQA also showed the same pattern of MMP reduction in the Caco-2 and Ishikawa cells (data not shown). Furthermore, the time-dependent changes in ROS and MMP matched the time course of cell death, as shown by the relevant time course data. These data indicate that CDDP and DQA exert cancer cell killing via mitochondrial dysfunction and ROS-induced cell death.

As previously reported, CDDP and DQA exert their antitumour effect through increased ROS generation and mitochondrial disruption [12, 14, 17]. Therefore, the present study investigated to what extent the ROS production was involved in cell death. When NAC (a powerful ROS scavenger) was administered to the cells prior to drug treatment, cell death was significantly reduced, indicating the critical role of enhanced ROS production in triggering cell death upon various CDDP and DQA treatments. It should be noted that in the Caco-2 cells, death attenuation with the combined therapy was more significant than that with the single treatment, which was in alignment with the highest ROS production level upon the combined treatment as compared to single treatment. Similarly, the attenuation effects in the Ishikawa cells were comparable among different treatments, indicating an equal (or non-treatment-specific) production level of ROS following the treatments, which was indeed the case as supported by the ROS data of the Ishikawa cells. Furthermore, the cell death attenuation by NAC was more effective for Ishikawa compared to Caco-2, indicating that ROS had a more important role in mediating cell death in Ishikawa. Those results collectively confirmed that baseline ROS levels could influence how cancer cells respond to the same treatment, as demonstrated by the Caco-2 and Ishikawa cells in this study. Moreover, our data highlight the fact that cancer cells with lower baseline levels of intracellular ROS would respond better to ROS-stimulating therapy by generating more ROS compared to cells with higher baseline levels of ROS. Hence, the excessive ROS induced by the treatment could be the main cause of cell death in cells with low baseline ROS, whereas different mechanisms might play a role in parallel with ROS in causing cell death in cells with high baseline ROS.

To gain further insight into the reason behind the differences in the baseline ROS level between the Caco-2 and Ishikawa cells, and the mechanisms underlying treatment-induced ROS production, the expression levels of three antioxidant genes, *SOD1*, *SOD2* and *CAT*, were analysed. Our data showed that the expression levels of these antioxidant genes were significantly lower in the naïve Caco-2 cells compared to Ishikawa. This could explain the differences in the baseline ROS level between the two cell lines, since cells producing less antioxidants are expected to have an inefficient ROS scavenging system, resulting in higher baseline ROS levels in those cells. Interestingly, all three genes were significantly upregulated only in the Caco-2 cells treated with DQA. Those results indicated that DQA treatment provoked an antioxidant response in the Caco-2 cells which remained silent upon exposure to CDDP. It is possible that in the Caco-2 cells, CDDP might have a direct impact on the expression levels of other antioxidants such as glutathione (GSH) and glutathione related enzymes (e.g. glutathione S-transferase/GST and glutathione peroxidase/GPX) which were not analysed in the present study. Potentially, CDDP exposure might also influence the expression and/or activity levels of certain transcription factors such as nuclear factor erythroid 2-related factor 2 (Nrf2) which regulates the expression of genes involved in the cellular antioxidant system and activating protein-1 (AP-1) which responds to cellular stresses including oxidative stress (reviewed in Ref.s [34–36]). As to the Ishikawa cells, the expression profiles of these antioxidant genes were less consistent compared to those of Caco-2. However, both *SOD1* and *CAT* were significantly downregulated in Ishikawa upon the combined treatment. The above results suggest that cancer cells with high baseline ROS levels might have relatively low expressions of antioxidant genes. However, upon ROS-stimulating treatment, those cells could upregulate their antioxidants significantly as a protective mechanism, rendering them more resistant to the treatment. On the other hand, cells with low baseline ROS levels might have saturated their antioxidant system and, therefore, could not defend themselves efficiently against ROS-stimulating compounds, as demonstrated by the significantly higher sensitivity levels of Ishikawa compared to those of Caco-2 in this study.

It should be emphasised that another advantage of using mitochondria-targeting cations in anticancer therapy, apart from the synergistic killing effect, is that those compounds preferentially enter mitochondria in cancer cells which generally have higher membrane potentials compared to those of mitochondria in normal cells. This allows cations to selectively target cancer cells and accumulate more rapidly in their mitochondria [15]. Our data comparing the prostate cancer (PC-3) and normal (PNT-2) cell lines confirmed the preferential targeting of cancer cells by DQA. Due to time and funding limitations, we were unable to carry out further in vivo work in the present study. However, we fully recognise that it is critical to test this in vitro phenomenon in vivo in order to properly evaluate the potential of DLCs in clinical practice. If proven, DLCs could offer cancer-specific therapy with significantly reduced side effects. MKT-077 was the first DLC to be tested in clinical trials which were terminated due to renal toxicity [15, 17]. Therefore, more suitable DLCs still need to be trialed.

## Conclusions

This study confirms that cisplatin and dequalinium exhibit different cytotoxic efficacy according to the baseline ROS levels within the cancer cells. To our knowledge, this is the first study showing that baseline ROS levels of cancer cells might be utilised to predict drug response. As measuring ROS in tissues is technically challenging, our data suggest that mtDNAcn could be a more efficient biomarker to indicate the response to mitochondria-targeting therapy. Our findings also suggest that combining conventional chemotherapy with mitochondria-targeting therapy enhances cell death and allows reduced doses of the conventional drug. As the above conclusions have been drawn based on limited in vitro work, further studies engaging a wider range of cell lines as well as in vivo experiments will be required to confirm the present findings. Nevertheless, this study has provided an insight into understanding the influence of intracellular ROS on drug sensitivity, and may lead to the development of new therapeutic strategies to improve anticancer drug efficacy.

## Supplementary information

**Additional file 1: Table S1.** Cell lines and their culture media employed in the study. **Table S2.** TaqMan assays used to measure the relative gene expression levels of the anti-oxidant genes. **Table S3. A.** List of primary antibodies used in west blotting. **B.** List of secondary antibodies used in west blotting. **Table S4.** The Measurement of Combination Index (CI). **Figure S1.** Increases in the intracellular ROS and mitochondrial superoxide levels in the Caco-2 and Ishikawa cells upon treatments during a 24-h period. **Figure S2.** Effects of CDDP and DQA at their IC50 concentrations, and a combination of both drugs at 1/2 IC50 concentrations, on cell viability in the Caco-2 (*a, b, c*) and Ishikawa (*d, e, f*) cells over a 24-h treatment period. **Figure S3.** Effects of CDDP and DQA at their IC50 concentrations, and a combination of both drugs at 1/2 IC50 concentrations, on mitochondrial membrane potential in the Caco-2 (*a, b, c*) and Ishikawa (*d, e, f*) cells over a 24-h treatment period. **Figure S4.** Decreases of mtDNA copy number of the Caco-2 (*a*) and Ishikawa (*b*) cells upon treatments at 24 h. **Figure S5** (*a*) Mitochondrial membrane potential of the PNT-2 and PC-3 cells. (*b*) Effects of DQA (10 µM) on mitochondrial membrane potential in the PNT-2 and PC-3 cells

## Data Availability

Data generated or analysed during this study are included in this article and its supplementary information document. Raw data used and/or analysed during the current study are available from the corresponding author on reasonable request.

## Abbreviations

CAT: Catalase
CDDP: Cisplatin
CI: Combination index
DLCs: Delocalised lipophilic cations
D-loop: Displacement-loop
DQA: Dequalinium / dequalinium chloride hydrate
GSH: Glutathione
IC50: The half maximal inhibitory concentration
MMP: Mitochondrial membrane potential
mtDNA: Mitochondrial DNA
mtDNAcn: Mitochondrial DNA copy number
NAC: N-Acetyl-L-cysteine
nDNA: Nuclear DNA
ROS: Reactive oxygen species
rRNA: Ribosomal RNA
SOD1: Cytoplasmic superoxide dismutase
SOD2: Mitochondrial superoxide dismutase
tRNA: Transfer RNA
