# Supplementary material for: ROS as a novel indicator to predict anticancer drug efficacy
Source: BMC Cancer. 2019 Dec 16;19:1224. doi: 10.1186/s12885-019-6438-y (PMC6916036; doi:10.1186/s12885-019-6438-y)
Supplement: Supplementary file 1 — Additional file 1: Table S1. Cell lines and their culture media employed in the study. Table S2. TaqMan assays used to measure the relative gene expression levels of the anti-oxidant genes. Table S3. A. List of primary antibodies used in west blotting. B. List of secondary antibodies used in west blotting. Table S4. The Measurement of Combination Index (CI). Figure S1. Increases in the intracellular ROS and mitochondrial superoxide levels in the Caco-2 and Ishikawa cells upon treatments during a 24-h period. Figure S2. Effects of CDDP and DQA at their IC50 concentrations, and a combination of both drugs at 1/2 IC50 concentrations, on cell viability in the Caco-2 (a, b, c) and Ishikawa (d, e, f) cells over a 24-h treatment period. Figure S3. Effects of CDDP and DQA at their IC50 concentrations, and a combination of both drugs at 1/2 IC50 concentrations, on mitochondrial membrane potential in the Caco-2 (a, b, c) and Ishikawa (d, e, f) cells over a 24-h treatment period. Figure S4. Decreases of mtDNA copy number of the Caco-2 (a) and Ishikawa (b) cells upon treatments at 24 h. Figure S5 (a) Mitochondrial membrane potential of the PNT-2 and PC-3 cells. (b) Effects of DQA (10 µM) on mitochondrial membrane potential in the PNT-2 and PC-3 cells [file 12885_2019_6438_MOESM1_ESM.docx]

**ROS as a Novel Indicator to Predict Anticancer Drug Efficacy**

**BMC Cancer**

Qian An, MD, PhD. Tel: +44 23 9284 2937. E-mail: [qian.an@port.ac.uk](mailto:qian.an@port.ac.uk)

Tarek Zaidieh. Tel: +44 23 9284 3633. E-mail: [tarek.zaidieh@port.ac.uk](mailto:tarek.zaidieh@port.ac.uk)

School of Pharmacy and Biomedical Sciences, University of Portsmouth, St Michael’s Building, White Swan Road, Portsmouth PO1 2DT, UK.

**Additional file 1**

**Table S1. Cell lines and their culture media employed in the study**

| **Cell line** | **RRID** | **Culture media** |
| --- | --- | --- |
| ***Ishikawa*** | CVCL_2529 | DMEM + FBS 5% |
| ***MDA-MB-231*** | CVCL_0062 | DMEM + FBS 10% |
| ***Caco-2*** | CVCL_0025 | DMEM + FBS 10% |
| ***PC-3*** | CVCL_0035 | RPMI 45% + HAMS'F12 45% + FBS 10% |
| ***PNT-2*** | CVCL_2164 | RPMI 45% + HAMS'F12 45% + FBS 10% |

**Table S2. TaqMan assays used to measure the relative gene expression levels of the anti-oxidant genes**

| **Gene** | **Ref Sequence** | **Source** |
| --- | --- | --- |
| *SOD1* | NM_000454(1) | Integrated DNA technologies |
| *SOD2* | NM_000636(3) | Integrated DNA technologies |
| *CAT* | NM_001752(1) | Integrated DNA technologies |
| *GAPDH* | NM_001686.3 | Life technologies |

**Table S3A. List of primary antibodies used in west blotting**

| **Protein** | **Antibody** | **Species** | **Dilution** | **Source** |
| --- | --- | --- | --- | --- |
| BCL-XL | MAB894 | Rabbit monoclonal | 1:500 | R&D systems, USA |
| Cytochrome *c* | MA5-11674 | Mouse monoclonal | 1:500 | ThermoFisher, USA |
| β-Actin | MAB8929 | Mouse monoclonal | 1: 2000 | R&D systems, USA |

**Table S3B. Table S3 B. List of secondary antibodies used in west blotting**

| **Antibody** | **Dilution** | **Source** |
| --- | --- | --- |
| Anti-rabbit HRP | 1:2000 | Dako, Denmark |
| Anti-mouse HRP | 1:5000 | Dako, Denmark |

**Table S4. The Measurement of Combination Index (CI)**

| **Caco-2 Cells** | | | |
| --- | --- | --- | --- |
| **Dose CDDP / µM** | **Dose DQA / µM** | **Effect** | **CI** |
| 5 | 90 | 0.66 | 0.99136 |
| 10 | 90 | 0.64 | 0.92967 |
| 50 | 90 | 0.60 | 0.94462 |
| 100 | 90 | 0.55 | 0.92323 |
| 250 | 90 | 0.45 | 0.90660 |
| 500 | 90 | 0.39 | 1.07629 |
| 1000 | 90 | 0.33 | 1.37327 |

| **Ishikawa Cells** | | | |
| --- | --- | --- | --- |
| **Dose CDDP / µM** | **Dose DQA / µM** | **Effect** | **CI** |
| 5 | 7.5 | 0.66 | 0.72731 |
| 10 | 7.5 | 0.59 | 0.63296 |
| 42.5 | 7.5 | 0.38 | 0.57506 |
| 50 | 7.5 | 0.34 | 0.55585 |
| 85 | 7.5 | 0.28 | 0.65842 |
| 100 | 7.5 | 0.25 | 0.66588 |
| 500 | 7.5 | 0.01 | 0.21071 |
| 1000 | 7.5 | 0.01 | 0.41721 |


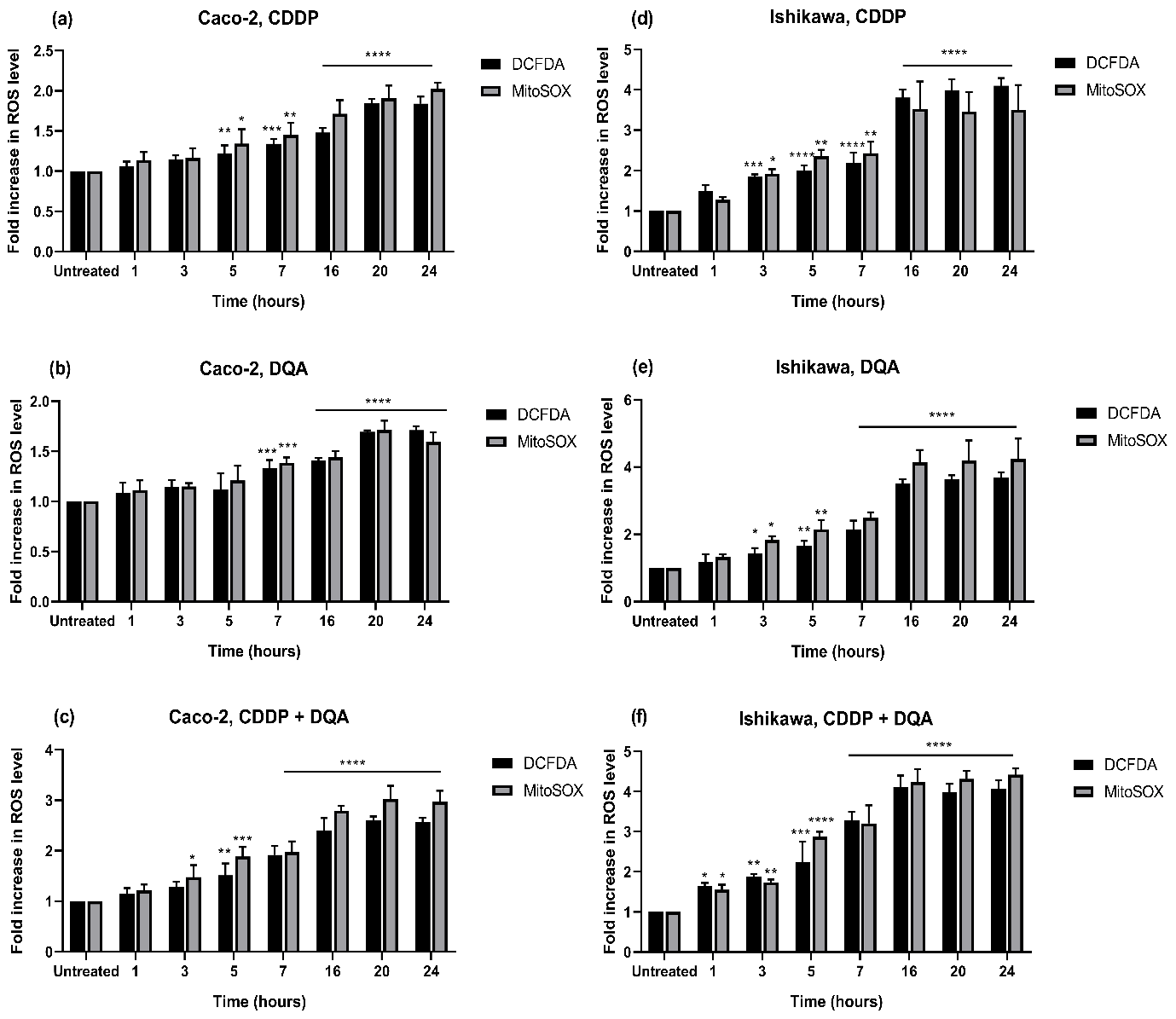


**Figure S1** Increases in the intracellular ROS and mitochondrial superoxide levels in the Caco-2 and Ishikawa cells upon treatments during a 24-hour period. The columns represent the fold changes of intracellular ROS (H2DCFDA) and mitochondrial superoxide (MitoSOX) respectively normalised against the untreated controls. Data are mean ± SEM (N=3 separate experiments); *p* values comparing treated and untreated cells were calculated using one-way ANOVA with Tukey multiple comparison post-hoc analysis; **p<*0.05, ***p<*0.01, ****p<*0.001 and *****p<*0.0001.

**Figure**
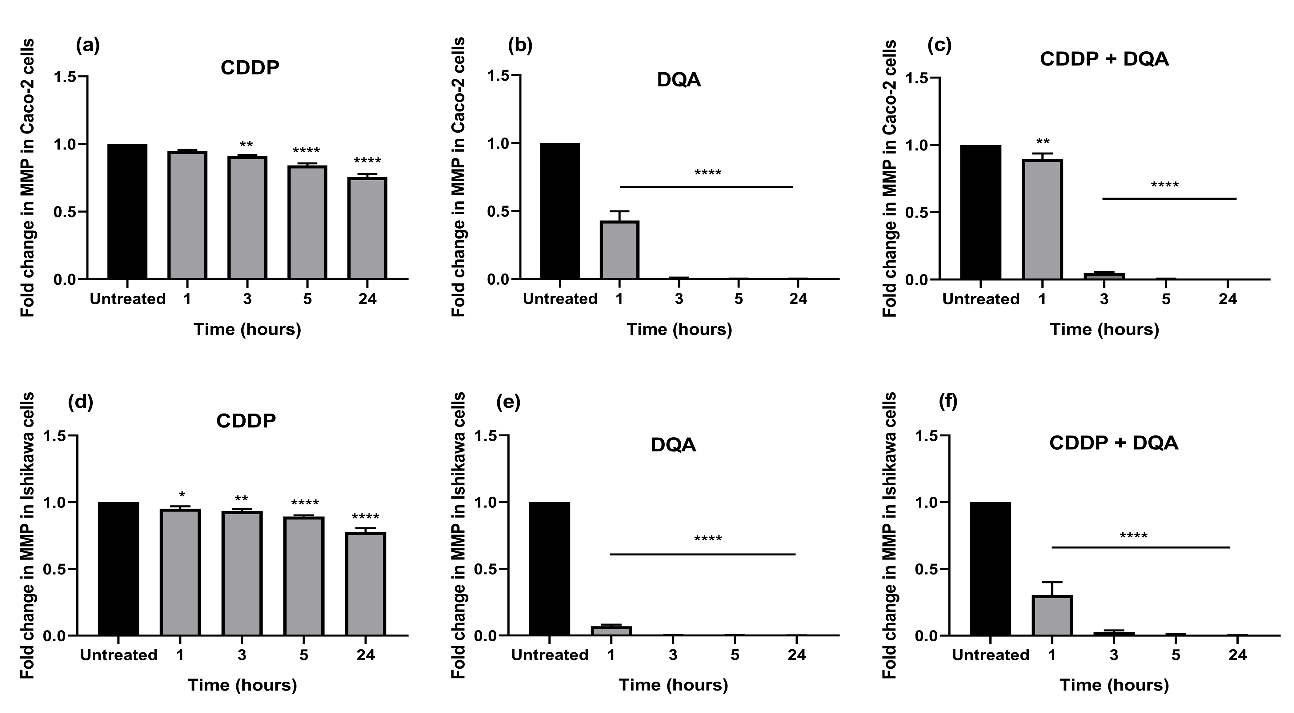

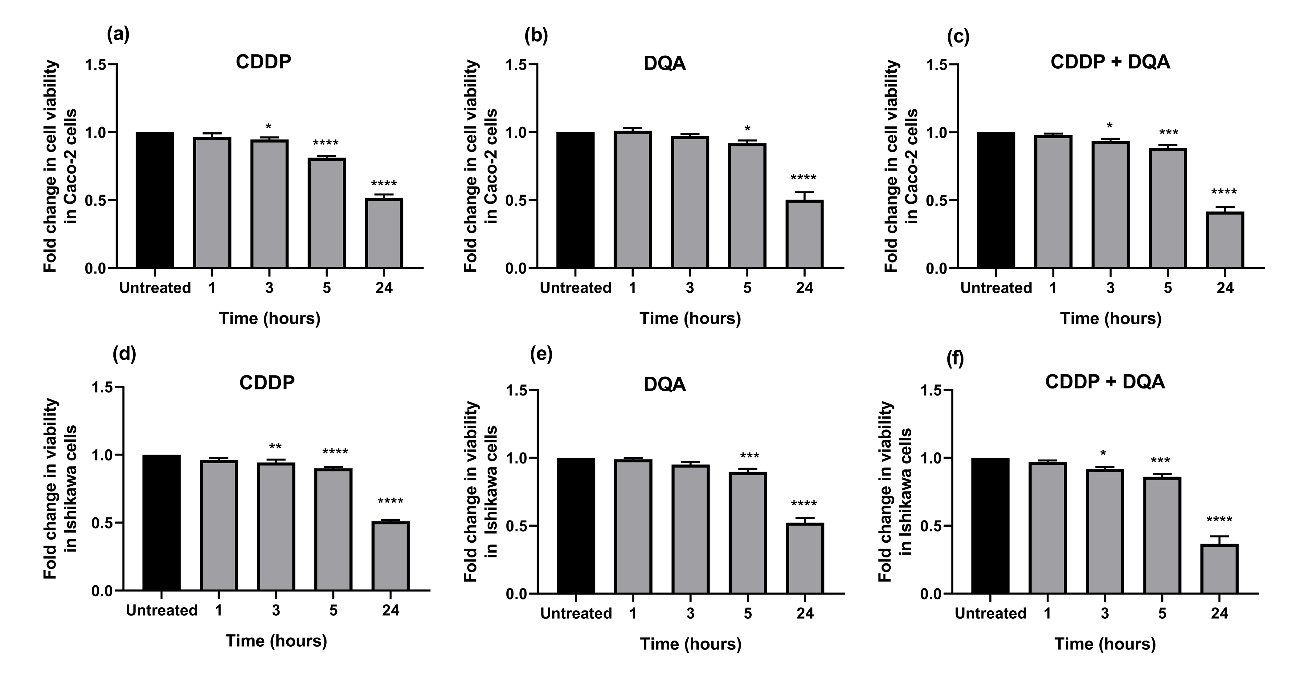
**S2** Effects of CDDP and DQA at their IC50 concentrations, and a combination of both drugs at 1/2 IC50 concentrations, on cell viability in the Caco-2 (*a, b, c*) and Ishikawa (*d, e, f*) cells over a 24-hour treatment period. The columns represent the fold changes of cell viability normalised against the untreated controls. Data are mean ± SEM (N=3 separate experiments); *p* values comparing treated and untreated cells were calculated using one-way ANOVA with Tukey multiple comparison post-hoc analysis; **p<*0.05, ***p<*0.01, ****p<*0.001 and *****p<*0.0001.

**Figure S3** Effects of CDDP and DQA at their IC50 concentrations, and a combination of both drugs at 1/2 IC50 concentrations, on mitochondrial membrane potential in the Caco-2 (*a, b, c*) and Ishikawa (*d, e, f*) cells over a 24-hour treatment period. The columns represent the fold changes of MMP normalised against the untreated controls. Data are mean ± SEM (N=3 separate experiments); *p* values comparing treated and untreated cells were calculated using one-way ANOVA with Tukey multiple comparison post-hoc analysis; **p<*0.05, ***p<*0.01, ****p<*0.001 and *****p<*0.0001.

**Figure**
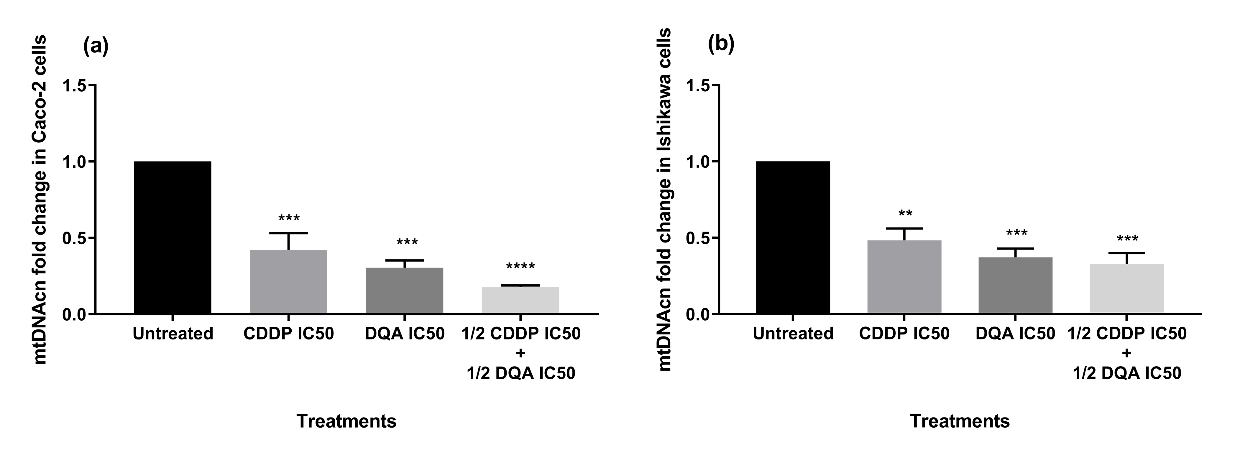
**S4** Decreases of mtDNA copy number of the Caco-2 (*a*) and Ishikawa (*b*) cells upon treatments at 24 hours. The columns represent the fold changes of mtDNAcn that were normalised against the untreated controls. Data are mean ± SEM (N=3 separate experiments); *p* values comparing treated and untreated cells were calculated using one-way ANOVA with Tukey multiple comparison post-hoc analysis; ***p<*0.01, ****p<*0.001 and *****p<*0.0001.

**
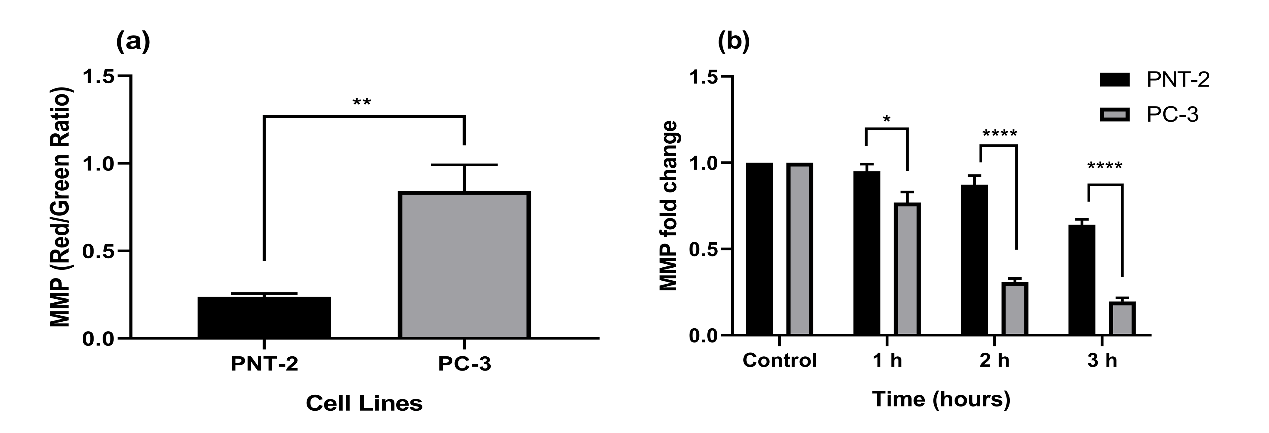
**

**Figure S5** (*a*) Mitochondrial membrane potential of the PNT-2 and PC-3 cells. The columns represent the red/green ratio of fluorescence intensity. Data are mean ± SEM (N=3 separate experiments); *p* values comparing PNT-2 and PC-3 was calculated using a two-tailed t-test; ***p<*0.01. (*b*) Effects of DQA (10 µM) on mitochondrial membrane potential in the PNT-2 and PC-3 cells. The columns represent the fold changes of MMP normalised against the untreated controls. Data are mean ± SEM (N=3 separate experiments); *p* values comparing the PNT-2 and PC-3 cells were calculated using a two-tailed t-test; **p<*0.05 and *****p<*0.0001.
